# Effect of the Addition of Nano-Silica and Poly(ε-caprolactone) on the Mechanical and Thermal Properties of Poly(lactic acid) Blends and Possible Application in Embossing Process

**DOI:** 10.3390/polym14224861

**Published:** 2022-11-11

**Authors:** Sanja Mahović Poljaček, Dino Priselac, Tamara Tomašegović, Urška Stanković Elesini, Mirjam Leskovšek, Mirela Leskovac

**Affiliations:** 1 Faculty of Graphic Arts, University of Zagreb, 10000 Zagreb, Croatia; 2 Faculty of Natural Sciences and Engineering, University of Ljubljana, Aškerčeva Cesta 12, 1000 Ljubljana, Slovenia; 3 Faculty of Chemical Engineering and Technology, University of Zagreb, 10000 Zagreb, Croatia

**Keywords:** PLA/PCL blends, nano-silica, mechanical properties, thermal properties, embossing

## Abstract

In this study, the mechanical and thermal properties of poly(lactic acid) (PLA) blends with an addition of poly(ε–caprolactone) (PCL) and fumed silica (SiO_2_) were evaluated to research the possibility of their use as relief printing plates for embossing processes. PCL and nano-silica were added to the PLA matrix at different concentrations. Morphological, thermal and mechanical analyses were performed to determine the properties and possible functional characteristics of the studied blends. SEM micrographs showed that unmodified PLA/PCL blends exhibit a morphology typical of incompatible blends with clearly visible spherical domains of dispersed PCL in PLA. In particular, the results of the hardness tests showed that the selected blends have the optimal hardness (between 65 SH D and 75 SH D) for use in the embossing process. The tensile tests showed that the addition of nano-silica to neat PLA and to the PLA/PCL blends 50/50 and 60/40 improved the mechanical properties of the blends, especially stiffness and toughness. The DMA results showed that the addition of smaller amounts of SiO_2_ can contribute to an increase in storage modulus, which is due to good dispersion and distribution of SiO_2_ in the matrix. DSC analysis showed that the addition of PCL to PLA polymer increased the thermal stability of PLA and that the addition of nano-silica increased the degree of crystallinity of PLA. The TGA results showed that the addition of nano-silica improved the thermal degradation behavior of the studied blends, especially for blends modified with 3 wt% nano-silica. The results show that it is possible to optimize the mechanical and thermal properties of the blends with the aim of using them in the embossing process.

## 1. Introduction

There is widespread interest in the use and production of biodegradable materials, as they could provide an alternative to conventional petroleum-based polymers and have a positive impact on the environment [1,2]. The use of biodegradable materials offers a number of advantages, i.e., less energy is required in the production process, the release of carbon dioxide is lower, the decomposition time is shorter and they can be easily recycled through an organic process, unlike fossil–based raw materials [3]. These are the reasons why a large number of industries are turning to the use of such materials, spreading awareness of sustainability and focusing on sustainable development.

Despite their advantages, biodegradable materials also have some limitations. Some of these are related to their manufacturing and composing process, and others to their price and properties. For example, the manufacturing process requires the use of expensive equipment, composing should be done by very specific disposal methods, biodegradable materials should not be mixed with non-biodegradable polymeric materials and if they are made from natural materials such as corn starch, the need for cultivated land must be increased.

Significant limitations also arise from the properties of biodegradable materials. In most cases, their original properties do not match their potential use, which is why biodegradable materials are rarely used as pure polymeric materials. More often, they are used in combination with other materials and fillers [4,5,6,7,8,9,10]. In this way, it is possible to adapt and finetune the necessary properties of the obtained materials for their potential application.

Nowadays, biodegradable materials can be found in the packaging industry, agriculture, medicine, pharmaceuticals, electronics, automotive, architecture and construction. They could also find application in the graphic arts industry, especially in the segment of printing plates, i.e., materials used for the printing process, where conventional polymers could be replaced by biodegradable materials.

It has already been published that polymer blends based on polycaprolactone (PCL) and polylactide (PLA) with the addition of coconut fiber could be used as printing plates for letterpress printing [11,12]. The aim of these studies was to propose biodegradable blends that could replace the conventional fossil-based polymers traditionally used for printing plates in relief printing [13]. It was found that it could be possible to adjust the properties of the prepared blends to produce a functional printing plate for relief printing. On the other hand, a newly prepared material had some shortcomings, mainly related to its mechanical and thermal properties, which are extremely important for the production of a functional printing plate. The results showed that the properties of the obtained materials could be optimized to some extent thanks to different concentrations of the components (PCL, PLA and coconut fibers).

However, due to the low melting temperature of the main component in the matrix (PCL, melting temperature *T_m_* = 60–65 °C [14]), the adjustment of the properties of the obtained mixtures was limited. For this reason, this research is focused on the preparation of a biodegradable blend based on PLA as the main component of the matrix, which has a higher melting temperature *T_m_* = 170 °C [15] compared to PCL, with the assumption that it should have better thermal properties to be used as a potential material for letterpress plates. When using PLA-based materials, the main weaknesses of these materials are their stiffness and brittleness.

This could be a significant disadvantage in the production of printing plates, since in the letterpress process the design must be transferred to the substrate by pressing the printing plate on the substrate’s surface. The pressure between materials makes it possible to damage the edge or surface of the printing plate, disrupting the design and causing uneven and irregular transfer of the design to the substrate. These and similar limitations of PLA have been previously reported in the literature, and the results presented showed that it is possible to overcome the limitations of PLA by blending it with a second polymer [6,7,16,17,18,19] or a nanoscale filler [15,20] to improve its properties.

In this research, PLA is used as the main component of the matrix for the preparation of blends with PCL, because the studies published on this topic have shown that the mixture of PLA and PCL can reduce the stiffness of the prepared blends and improve the toughness [4,16,17,21,22]. Since PLA and PCL are relatively immiscible polymers [7,21,23], nanoscale silica was added to PLA/PCL blends to increase their miscibility and thus optimize the properties of the resulting blends. Nanoscale silica has been used in different studies as a filler for improving the miscibility and mechanical and thermal properties of PLA and PCL blends [24,25,26,27,28].

## 2. Materials and Methods

### 2.1. Materials

PLA was supplied by InegoTM 3251D, Nature Works LLC, Plymouth, MN, USA, and PCL was supplied by Capa 6800, Perstorp, Warrington, UK. PLA is stiff and brittle below its glass transition temperature (*T_g_* = 50–60 °C) and with a melting temperature of *T_m_* = 170 °C [15]. Tensile strength of neat PLA is 60 MPa and tensile elongation equals 3.5%. The glass transition and the melting temperatures of PCL are −60 °C and 60–65 °C, respectively [14]. The tensile strength of neat PCL is 20 MPa and tensile elongation equals 800%. The fumed silica (Aerosil^®^200) was kindly supplied by Evonik (Hanau, Germany), CAS No. 112945–52–5, with average particle size of 12 nm. They were used as received without any pre-treatment.

### 2.2. Preparation of PLA/PCL Blends

**Figure 1:**
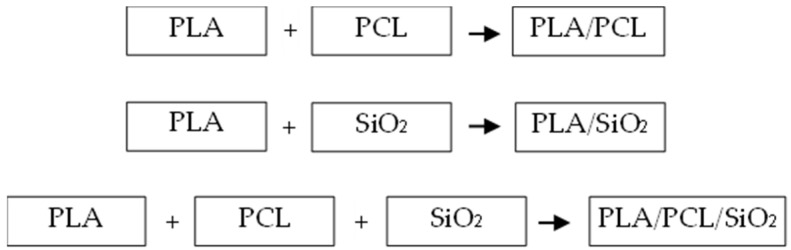
Schematic flow diagram of blending PLA with PCL and SiO_2_.

The basis for the preparation of polymeric biodegradable matrix was PLA, while PCL was added to PLA in a certain ratio. The weight ratio of the components was set from PLA/PCL 100/0 to 90/10, 80/20, 70/30, 60/40 and 50/50. Nano-silica was added to the blends at concentrations of 1 wt% and 3 wt%. Measurements were performed on PLA/PCL blends without and with added nano-silica. Figure 1 shows a schematic flow diagram of preparation stages of PLA/PCL blends without and with added nano-silica.

The materials were blended in the Brabender^®^ internal mixer for 5 min at a temperature of 190 °C and cut into pieces. The cut pieces were molded into plates with dimensions of 100 mm × 100 mm × 1 mm using a hydraulic press for seven minutes (two minutes of preheating and five minutes of pressing) at a temperature of 190 °C and a pressure of 16 MPa. After the cooling process, the samples were ready for analysis.

### 2.3. Characterization Methods

The scanning electron microscope (SEM) was used to observe the surface morphology of the mixed materials in produced blends in cross-sectional view. A scanning electron microscope JSM–6060LV (Jeol, Tokyo, Japan) was used. Before the imaging, the samples were coated with a layer of gold to achieve the electro-conductivity of the samples (by high vacuum evaporation).

The hardness of the produced blends was measured using the Zwick Roell 3130 Hardness Tester (ZwickRoell Group, Ulm, Germany). The Shore D method was used, commonly used in measuring the hardness of hard plastics and rubber which operates according to the standards ISO 48–4, ASTM D2240, ISO 868 and NFT 51109. Observing the hardness values of the material used for printing plates is of great importance for evaluating their functional properties in the embossing process. The hardness requirements for printing plates can range from 60 SH D to 75 SH D, depending on the material used as the printing substrate. The procedure was carried out by placing four samples (at least 4 mm high) in the instrument. Shore hardness evaluation is based on measuring the elastic bounce of a needle of a certain mass when it is dropped from a given height onto a test material, measuring the bounce height. The bounce height is proportional to the hardness of the material. The hardness value, expressed in SH D, is displayed on the digital screen. The measured hardness of the produced samples is presented as the average of ten measurements.

Tensile tests were performed to determine the mechanical properties of the produced blends and to determine the influence of the composition of the blends on the material’s strength and deformation behavior until break. Before performing tensile testing of PLA and polymer blends, samples with dimensions of 1 cm × 10 cm were cut from the plate obtained by pressing. The cut samples were placed in universal testing machine Zwick 1445 (ZwickRoell Group, Ulm, Germany) in uniaxial tension mode at 23 °C and 65% relative humidity. The measurement was carried out at a crosshead speed of 10 mm/min and an initial length between clamps of 50 mm. Tensile strength (*σ*), Young’s modulus (*E*), strain at break (*ε_b_*) and work to break (*W*) were measured for each sample, and the measurement was carried out five times in order to determine the mean value of the mechanical properties.

Dynamic mechanical analysis (DMA) was performed using a Q800 DMA analyzer (TA Instruments, New Castle, DE, USA). DMA is an analytical technique for measuring the mechanical and viscoelastic properties of materials and is well suited for evaluating the compatibility of the observed blends. In DMA analysis, the elastic and viscous response of the material under study can be measured as a function of temperature, time, or frequency by applying an oscillating force. Modulus as a function of time or temperature is measured and provides information on phase transitions. Measurements were performed in a dual cantilever bending mode on materials with a length of 35 mm at an oscillation frequency of 10 Hz, an oscillation amplitude of 10 nm and a temperature step (ramp) of 3 °C/min in the range of −80 °C to + 40 °C.

Thermal behavior and crystallization of the samples was measured by differential scanning calorimetry analysis (DSC). Measurement was carried out on a Mettler Toledo DSC 823e (Columbus, OH, USA). The samples of approximately 10 mg weight were placed in an aluminum pan and heated from −90 °C to 200 °C. Tests were performed under an inert nitrogen atmosphere at a flow rate of 50 cm^3^/min and cooled by an Intracooler at a heating/cooling rate of 10 °C/min. The DSC thermograms with the glass transition temperature (*T_g_*), melting points (*T_m_*), crystallization temperature (*T_cc_*), enthalpy of cold crystallization (Δ*H_cc_*) and enthalpy of melting (Δ*H_m_*) were recorded during the second heating cycle. Crystallization temperature (*T*_c_) and enthalpy of crystallization (Δ*H_c_*) of neat polymers and polymer blends were recorded for the cooling cycle.

The degrees of crystallization of PLA phase (*X_c (PLA)_*) and PCL phase (*X_c (PCL)_*) were calculated according to the following Equations (1) and (2) [29]:

(1) $$X_{c\;\left(\mathrm{PLA}\right)}=\frac{\left(\Delta H_{m}-\Delta H_{cc}\right)}{\Delta {H^{0}}_{m}\times w}\times 100$$

where *X_c_*_(PLA)_ is the percentage of crystallinity of PLA; Δ*H_m_* and Δ*H_cc_* are the enthalpy of fusion and cold crystallization of PLA [J·g^−1^]; Δ*H*^0^*_m_* is the enthalpy of fusion of 100% crystalline PLA (where the enthalpy of fusion of 100% PLA is 106 J·g^−1^ [29]) and *w* is the mass fraction of PLA.

The fraction of PCL crystallinity (*X_c_*_(PCL)_) was calculated from the enthalpy of fusion and cold crystallization according to Equation (2):

(2) $$X_{c\;\left(\mathrm{PCL}\right)}=\frac{\Delta H_{m}}{\Delta {H^{0}}_{m}\times w}\times 100$$

where *X_c_*_(PCL)_ is the percentage of crystallinity of PCL; Δ*H_m_* is the specific enthalpy of fusion [J·g^−1^]; Δ*H*^0^*_m_* is the enthalpy of fusion of 100% crystalline PCL polymer (where the enthalpy of fusion of 100% PCL is 139.3 J·g^−1^ [30]) and *w* is the mass fraction of PCL.

Thermogravimetric analysis (TGA) was carried out in order to observe the thermal stability and thermal degradation of observed blends using a TA Instruments Q500 (New Castle, DE, USA). The change in mass of samples was measured during controlled temperature change and under defined atmosphere conditions. Approximately 10 mg of PLA/PCL blends with and without addition of nano-silica were heated from 25 °C to 900 °C in an inert nitrogen atmosphere with a flow rate of 60 cm^3^/min.

## 3. Results

### 3.1. Morphology of Samples

**Figure 2:**
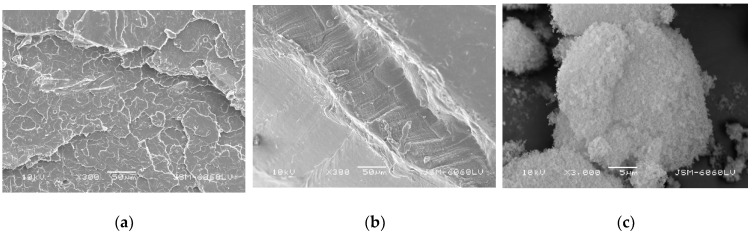
SEM micrographs of fracture surfaces of: (**a**) neat PLA, (**b**) PCL (mag. 300×) and (**c**) silicon–dioxide (fumed silica) as received (mag. 3000×).

Figure 2 shows microscopic images of the fracture surfaces of the original polymer components PLA and PCL and the microscopic image of the fumed silica. Figure 2a shows the surface of the pure PLA. It can be seen that the surface is uniformly textured and filled with irregular and sharp repeating structures. The fracture surface is typical of a brittle material, suggesting the absence of plastic deformation, which is consistent with one of the main characteristics of a neat PLA. On the other hand, the microscope image of a neat PCL shows no traces of sharp and brittle structures; the surface is distorted and irregular, suggesting the existence of a certain degree of elasticity in the polymer structure (Figure 2b). These observations are consistent with the results found in the literature [19,28,31]. Figure 2c shows nanoparticles of fumed silica at 3000× magnification.

According to the technical specification, individual silica particles are about 12 nm in size. Furthermore, it is known that nano-silica tends to form larger, micrometer-sized agglomerates composed mainly of particles stabilized by hydrogen bonding and electrostatic interactions and having a large specific surface area and hydrophilicity [32] which are clearly visible in the image.

**Figure 3:**
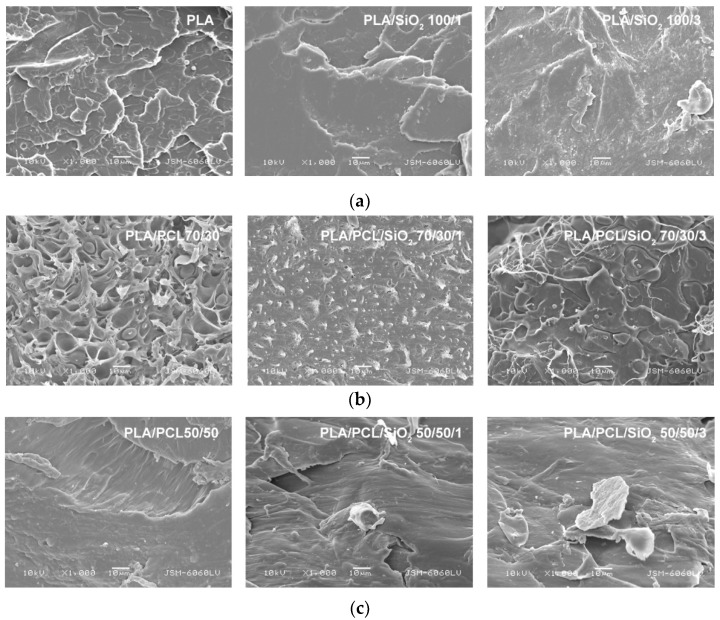
SEM micrographs of fracture surfaces of: (**a**) neat PLA, PLA/SiO_2_ 100/1, PLA/SiO_2_ 100/3; (**b**) PLA/PCL 70/30, PLA/PCL/SiO_2_ 70/30/1, PLA/PCL/SiO_2_ 70/30/3 and (**c**) PLA/PCL 50/50, PLA/PCL/SiO_2_ 50/50/1, PLA/PCL/SiO_2_ 50/50/3 (mag. 1000×).

The morphology of PLA/PCL blends in unmodified and nano-silica compatibilized form can be seen in the images from SEM (Figure 3). SEM microscopic images of fractured surfaces of PLA without and with the addition of 1 wt% and 3 wt% nano-silica show that the changes in the surface structure are caused by the addition of nano-fillers and are mainly visible in the form of a reduction in the irregular and sharp repeating structures (Figure 3a). Figure 3b (left image) shows the structure of the PLA/PCL 70/30 blend without nanoparticles. Two distinct phases can be seen, with spherical domains of dispersed PCL in PLA, indicating that PLA and PCL are not compatible. By adding nano-silica to the PLA/PCL 70/30 blend, it can be seen that the fracture surface has changed and the spherical elements have been reduced, indicating better interactions between the components at the interface with the polymer matrix. These changes are clearly visible on the microscopic images of the PLA/PCL/SiO_2_ blend 70/30/3. The effect of the addition of nano-silica in the PLA/PCL 50/50 blend can be seen in Figure 3c. It can be said that the fractured surface of the blend with equal composition of PLA and PCL and with the addition of nano-silica shows an irregular structure with separated details representing unevenly distributed components in the blend. Separated structures indicate the appearance of agglomerates specific to nano-silica. It has been previously published that nano-silica with a larger surface area has a high degree of dispersion in the composites with a higher amount of PLA. These results can be seen in the following SEM images [25].

### 3.2. Mechanical Properties

**Figure 4:**
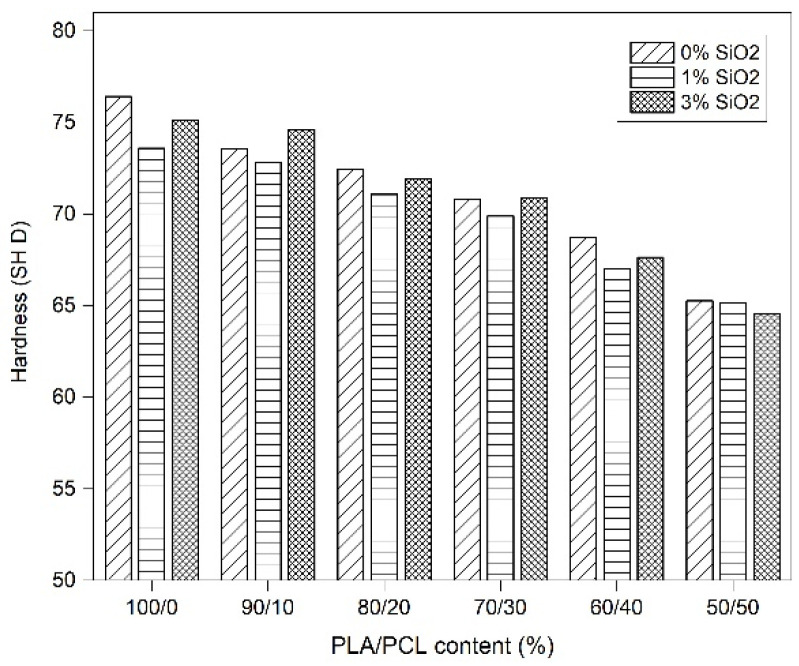
Hardness of PLA/PCL/SiO_2_ blends.

The mechanical properties of the blends were measured to determine the influence of the nano-silica filler on the neat PLA and the PLA/PCL blends. The results of the hardness test are shown in Figure 4. It can be seen that the addition of PCL to PLA causes a decrease in hardness in the observed blends. These results were expected due to the fact that blending of PCL into the PLA matrix can improve the toughness of PLA/PCL, which is consistent with the previously published research results [4,16,17,21,33]. The measured hardness of neat PLA is 76.42 SH D. The addition of PCL to the PLA continuously decreases the hardness value to 65.25 SH D for the blend PCL/PLA 50/50.

It can also be seen that the hardness of the blends changed slightly with the addition of nano-silica. The highest hardness was observed in the neat PLA without SiO_2_ (76.5 SH D). Considering that the studied blend is intended for use in embossing, where the brittleness of pure PLA is not desirable, this increase in material hardness may affect the ease of breaking the printing plate during use. Other samples, especially PLA/PCL 70/30 and PLA/PCL 90/10 with an addition of 3 wt% SiO_2_, showed higher hardness values compared to the same blends without SiO_2_. These results could be attributed to the reinforcing effect of nano-silica in the observed blends, which increases the molecular interaction between PLA and PCL and consequently improves the hardness of the blends [34]. It can be said that 3 wt% silica has a greater effect on the hardness value than 1 wt%. As mentioned earlier, the hardness requirements for printing plates used in embossing can range from 60 SH D to 75 SH D, so it can be said that all the observed materials have optimum hardness values for the observed purpose, but finetuning the functional properties of the observed blends can be done by adding 3 wt% SiO_2_ in PLA/PCL blends [9,21,35].

The results of tensile tests are presented in Table 1 where average and standard deviation (SD) values are reported. The results of tensile strength (*σ*), Young’s modulus (*E*), strain at break (*ε_b_*) and work to break (*W*) for all samples can be seen. Tensile measurements showed that there were significant differences in mechanical properties of neat PLA and PLA/PCL blends without and with the addition of SiO_2_ nanoparticles. Neat PLA showed a low strain at break (*ε_b_* = 2.78%) and high value of Young’s modulus (*E* = 1590.4 MPa), typical for glassy polymers, which limits its use for deformation applications [36,37,38]. It can be seen that the initial high Young’s modulus and tensile strength of PLA decrease continuously and almost linearly as a function of the composition of the added PCL. It was expected that with the increase of the volume fraction of PCL, the modulus and tensile strength of PLA/PCL blends would decrease because PCL has good elastic properties and allows high deformation without fracture [30,37,39]. Moreover, the strain at break exhibits low values in all samples, which is probably due to the high amorphous content in the PLA component and the immiscibility of the two polymers, indicating the need to use nano-fillers as a potential compatibilizer [40]. Certain increasement of strain at break was observed when PCL was added to the PLA, indicating a reduction in the brittleness of the observed blends.

On the other hand, it can be seen that the addition of 1 wt% and 3 wt% of silica cause an increase in the elastic modulus for all samples, the neat PLA and the PLA/PCL blends, probably due to the reinforcing effect of the nanoparticles, which can lead to a qualitative improvement in the strength and stiffness of the observed materials. Moreover, the values of tensile strength of blends with silica are lower compared to PLA/PCL blends without silica. These lower values of tensile strength of PLA/PCL blends occurring with the addition of SiO_2_ could be attributed to the less adequate adhesion properties between PLA, PCL and nano-silica, resulting in poor interfacial interaction between the materials in the matrix.

The exception is neat PLA and PLA/PCL blends (50/50 and 60/40), where the addition of nano-particles results in a slight increase in tensile strength and higher values of strain at break. This can be attributed to the possible reduction in the size of PCL particles during the blending process, which occurs when a nano–filler is added to the blends. Fortelny et al. have published that this effect occurs in PLA/PCL blends with a higher content of dispersed phase and that it could cause the stabilization of the blend properties [41]. In conclusion, one can say that the addition of nano-silica to neat PLA and to the PLA/PCL 50/50 and 60/40 blends causes an improvement in the mechanical properties of the blends, especially stiffness and toughness. These results are of great importance for the use of these biodegradable blends as material for printing plates.

### 3.3. Thermal Properties

**Figure 5:**
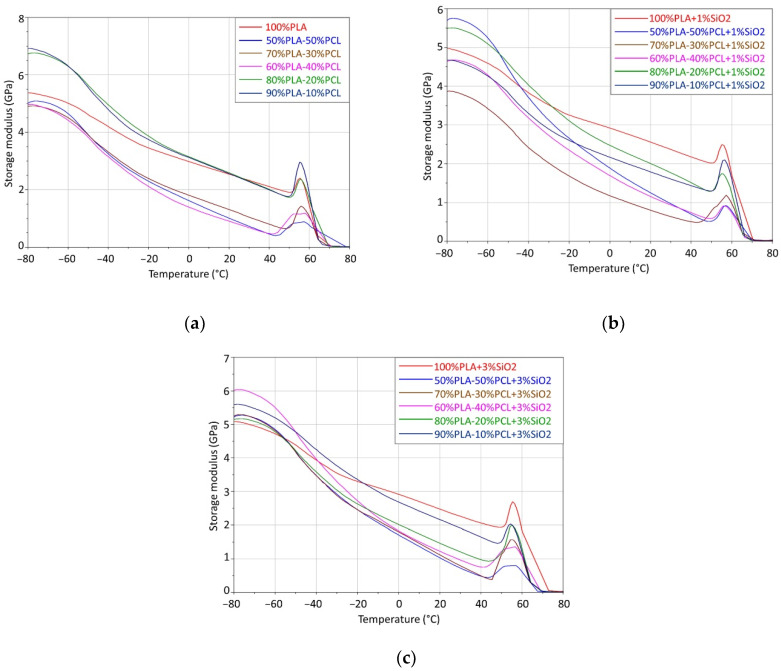
Storage modulus of blends: (**a**) PLA/PCL; (**b**) PLA/PCL and 1 %wt SiO_2_ and (**c**) PLA/PCL and 3 %wt SiO_2_.

The results of DMA testing are presented in Figure 5 and in Table 2. The results show two relaxation regions with two glass transition temperatures (*T_g_*). The glass transition temperature at higher temperatures belongs to PLA and at lower temperatures belongs to PCL. The DMA storage modulus displays two relaxation areas for the blend samples. The first transition area (from −60 to −20 °C) with first relaxation transition point at approx. 50 °C (corresponding to *T_g_* of PCL component) and the second transition area (from −60 to−20 °C) with the second transition point at approx. 60–70 °C (corresponding to *T_g_* of PLA component). This conclusion demonstrates the immiscibility of PLA and PCL.

The PCL spherical structure formed in the PLA matrix represents a weak point where stress concentration occurs, which contributes to the reduced elastic modulus *E*’, which occurs with an increase in the proportion of PCL in the PLA matrix (Figure 5 and Table 2) [17].

In glassy state, by adding SiO_2_ nanoparticles to the mixture, the storage modulus increases, except for the samples with equal portions of PLA and PCL (50/50/1 and 50/50/3) due to the increased interfacial area between SiO_2_ and the polymer matrix. The addition of smaller amounts of SiO_2_ can contribute to an increase in the storage modulus caused by good dispersion and distribution within the matrix. On the other hand, with an increase in the proportion of nano-silica in the mixture, the strengthening effect caused by the addition of SiO_2_ decreases (especially seen when observing the modules at room temperature, i.e., 20 °C). The reason for this is the aggregation of nanoparticles, which causes their poor dispersion and distribution within the mixture and leads to greater surface irregularities and the creation of voids within the structure. Thus, there is a reduction of the interfacial area between SiO_2_ and the mixture, which results in a weak interaction between nano-silica and the blends [24,42].

By adding a nano-silica into the blend, the glass transition temperature increases due to a decrease in the mobility of the entire volume of the polymer, and this decrease in mobility can be limited only to the polymer chains located in the area just a few nanometers from the SiO_2_ surface. A decrease in the glass transition temperature may occur due to the weak interaction between SiO_2_ and probably due to formation of the free volume spots between the polymer chains, caused by the presence of SiO_2_ particles [43]. The convergence of the two glass transition temperatures occurs when SiO_2_ acts as a weak compatibilizer between PLA and PCL.

In the glassy region, the values of the storage modulus of blends vary between 3.9–5.7 GPa, visible in Table 2. Neat PLA has the storage modulus 5.37 GPa and by addition od PCL content, it has been decreased to 3.87 for the blend containing 30 wt% of PCL and 1%wt of nano-silica (PLA/ PCL/SiO_2_ 70/30/1). With the addition of PCL, obviously the storage modulus of blends reached lower values (4.91 GPa and 5.02 GPa for the blend containing 30 wt% of PCL and 50 wt% of PCL, respectively). The addition of SiO_2_ in the PLA/PCL blends lowers the storage modulus when compared to the neat PLA (except for the blend containing 50 wt% of PCL and 1%wt of nano-silica (PLA/PCL/SiO_2_ 50/50/1), where the storage modulus has increased compared to neat PLA).

When the temperature is increased to room temperature, the storage modulus of all samples decreases due to higher molecular mobility and reduced intermolecular forces [44]. The elasticity of all samples progressively drops due to the formed and increased plastic component in the samples (induced by the heat).

The thermal stability of the PLA/PCL blends was studied with DSC analysis. It was performed to understand the crystallization and melting behavior of the PLA/PCL blends with and without addition of nano-silica. The glass transition temperature (*T_g_*), melting points (*T_m_*), temperature of cold crystallization (*T_cc_*), enthalpy of cold crystallization (Δ*H_cc_*), melting enthalpy (Δ*H_m_*), crystallization temperature (*T_c_*) and enthalpy of crystallization (Δ*H_c_*) of neat polymers and polymer blends are listed in Table 3, Table 4 and Table 5.

The results of the second heating cycle showed that glass transition temperature of PCL is in the interval from −70 to −40 °C, and melting temperature of PCL is in the interval from 55 to 65 °C (Table 3). Cold crystallization temperature of PLA is around 100 °C and recrystallization of PLA is in the area around 155 °C. Melting temperature of PLA is in the region of around 170 °C. The glass transition temperature of PLA was not observed in the samples with PCL because this temperature corresponds to the melting temperature of PCL, so the two regions overlapped. In samples without PCL, the glass transition temperature of PLA is around 59 °C. The melting temperature of PLA has a slight increase when adding SiO_2_ nanoparticles to the mixture. The addition of SiO_2_ nanoparticles in the amount of 1% leads to a decrease in the glass transition temperature of PCL, i.e., to an increase in samples with the addition of SiO_2_ nanoparticles in the amount of 3%. The only exception is the sample PLA/PCL/SiO_2_ 90/10/3, where there is a decrease in the glass transition temperature of PCL compared to the same mixture without and with 1% addition of SiO_2_ nanoparticles. The lowest value of the glass transition temperature of PCL was observed with the sample PLA/PCL/SiO_2_ 80/20/1, and the highest with the sample PLA/PCL 90/10. The thermal transition temperatures in these blends are specific to pure PLA and pure PCL, indicating their poor miscibility.

On the other hand, it can be seen that the degree of crystallinity of PLA in the blends (Table 5) is increased with the addition of PCL and the addition of nano-silica. The higher amount of PCL in the PLA/PCL blends cause the increase in PLA crystallinity degree, i.e., it promotes the crystallization of PLA due to the nucleation effect of the secondary phase. These results are in agreement with the previously published results on the nucleation of PLA in the presence of PCL [12]. The only exception is the sample PLA/PCL 60/40, where the addition of PCL in the blend caused a decrease of degree of crystallinity of PLA. Furthermore, one can see that addition of nano-silica causes the same effect for most of the samples, it increases the PLA crystallinity degree and interferes with the crystallization of PLA in the PLA/PCL blends. The exception is sample PLA/PCL 80/20, where the addition of silica caused a decrease of degree of crystallinity of PLA. From the results of the percentage of crystallinity of PCL, it is visible that *X_c (PCL)_* is decreased by addition of nano-silica, what means that nano-silica interferes with the crystallization of PCL.

The results of the cooling cycle showed that PLA crystallization temperature occurs in the area from 86 °C to 93 °C. The exception is the sample PLA/PCL 50/50 where this temperature is 116 °C and the sample PLA/PCL 80/20 where it is 83 °C. The crystallization temperature of PCL is between 22 °C and 28 °C.

**Figure 6:**
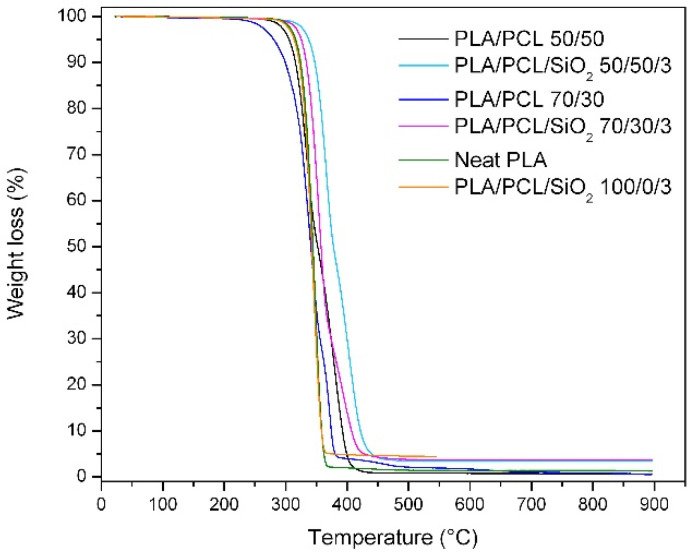
TG curves of neat PLA, PLA/SiO_2_ 100/3, PLA/PCL 70/30, PLA/PCL/SiO_2_ 70/30/3. PLA/PCL 50/50 and PLA/PCL/SiO_2_ 50/50/3.

TGA measurements were performed to further determine thermal stability and thermal degradation behavior of the PLA/PCL blends without and with addition of SiO_2_. TG curves for selected blends are shown in Figure 6 and in Table 6, where the values of temperature at the beginning of decomposition (*T_onset_*), temperatures at 5 and 50% mass loss, temperatures at the maximum rate of decomposition (*T_max_*) of the PLA/PCL blends and the residual mass at 600 °C (R_600 °C_) are presented. One can see that curves of neat PLA and PLA/PCL blends correspond to the amounts of individual polymers mixed into the blends. Neat PLA curve exhibited single-stage degradation mechanism and in compositions with the certain amount of PCL, the degradation of blends takes place in two degradation stages, because PLA and PCL are mutually immiscible polymers [21,44,45]. The maximum degradation temperature that corresponds to the highest decomposition rate for neat PLA and neat PCL is around 349 °C and 432 °C [12], respectively. In the PLA/PCL blends, the first stage degradation corresponds to the degradation of PLA, and the second to the degradation of PCL, which correspond to a higher thermal stability of PCL compared to PLA [7,46,47]. It has already been published that the addition of PCL components in PLA blends shifts the thermal degradation rate to higher temperatures, but according to the present results, such occurrence could be different. It can be seen that for blends without nano-silica, a slight decrease in the maximum decomposition temperature is visible with the addition of PCL in PLA. The decreasing decomposition temperature could be the result of deployment of PCL in the PLA/PCL matrix, especially because these polymers are immiscible, which affects the structure and thermal resistance of the blend, but it could also be a result of the formation of free radicals that initiate thermal degradation at a lower temperature [39,48]. The temperature required for the decomposition of 5% of the mass of the sample is reduced for PLA/PCL 70/30 and PLA/PCL 50/50, and also the temperature at 50% loss of the mass of the PLA/PCL 70/30. On the other hand, it is slightly increased for blend PLA/PCL 50/50.

The presence of nano-silica in the neat PLA caused a slight decrease in maximum temperature degradation rate, it is shifted from 349°C for neat PLA to 345 °C for PLA/SiO_2_ 100/3. Addition of nano-filler in the PLA/PCL blends showed an increase of the maximum temperature degradation of PLA and PCL. In the PLA/PCL/SiO_2_ 70/30/3 blend, *T_max_* for PLA is shifted from 341 °C (without nano-silica) to 355 °C (with nano-silica) and in the PLA/PCL/SiO_2_ 50/50/3 blend from 335 °C (without nano-silica) to 367 °C (with nano-silica), indicating better thermal stability of PLA component in observed blends. *T_max_* for PCL is shifted from 372 °C (without nano-silica) to 395 °C (with nano-silica) in PLA/PCL/SiO_2_ 70/30/3 blend and from 380 °C (without nano-silica) to 403 °C (with nano-silica) in PLA/PCL/SiO_2_ 50/50/3 blend. These results showed that addition of nano-silica improved the thermal degradation behavior of observed blends, especially for blends modified with 3 wt% of nano-silica. The improvement in thermal stability could be explained by the interaction between well-dispersed nano-particles and the polymer matrix. The addition of 3 wt% SiO_2_ increased the thermal stability of the samples. Observing the residue after decomposition, it is visible that its increase is caused by the addition of SiO_2_, which makes up the majority of the residues.

**Figure 7:**
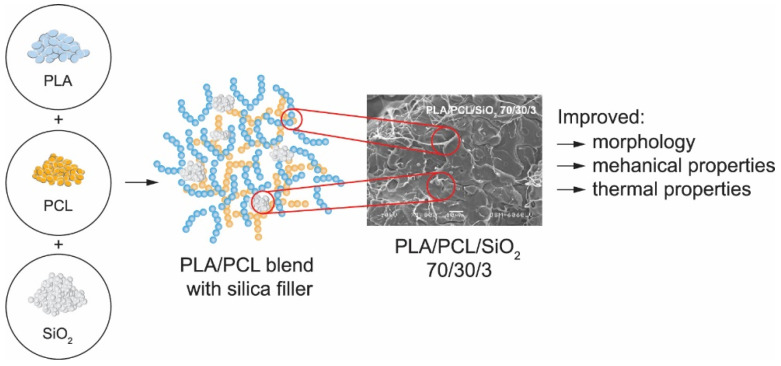
Influence of the silica nano-filler on the properties of the observed PLA/PCL blends.

The displayed results can be shown in a schematic diagram to illustrate the influence of the silica nanofiller on the properties of the observed PLA/PCL blends (Figure 7).

## 4. Conclusions

In this research, the mechanical and thermal properties of PLA and PCL blends with an addition of fumed silica (SiO_2_) as a nano-filler were evaluated to investigate the possibility of their use as relief printing plates for embossing processes. The main component of the matrix was PLA: PCL and nanoscale silica were added to PLA in different concentrations. The aim of the research was to increase the miscibility of PLA/PCL/SiO_2_ blends and thus optimize the functional properties of produced blends.

The results showed that it is possible to optimize the mechanical and thermal properties of the blends. It was shown that the addition of nano-silica changed the morphology of observed blends. Specifically, the results of the hardness tests showed that the selected blends have the suitable hardness (between 65 SH D and 75 SH D) for use in the embossing process. The highest hardness was observed in the neat PLA without SiO_2_ (76.5 SH D), which was considered too high for use in embossing. Other samples, particularly PLA/PCL 70/30 and PLA/PCL 90/10 with an addition of 3 wt% SiO_2_, showed optimal hardness values (about 70 SH D). The tensile tests showed that the addition of nano-silica to neat PLA and to the PLA/PCL 50/50 and 60/40 blends improved the mechanical properties of the blends, especially stiffness and toughness. The DMA storage modulus shows two relaxation areas for the blend samples, indicating the immiscibility of PLA and PCL. The results have shown that the addition of smaller amounts of SiO_2_ can contribute to an increase in the storage modulus caused by good dispersion and distribution within the matrix. DSC analysis showed that the addition of PCL to PLA polymer increased the thermal stability of PLA, and that the addition of nano-silica increased the degree of crystallinity of PLA and hindered the crystallization of PLA in the PLA/PCL blends. TG analysis proved that PLA and PCL are immiscible polymers by showing the two stages of decomposition of PLA/PCL blends. The TGA results showed that the addition of nano-silica improved the thermal degradation behavior of the observed blends, especially for blends modified with 3 wt% nano-silica. The results presented suggest that the use of PLA/PCL/SiO_2_ blends as materials for the embossing process has great potential. Further research should demonstrate the functional properties of the prepared blends in embossing a design onto a paper substrate through produced printing plates.

## Figures and Tables

**Table 1:** Results of the tensile tests for neat PLA and PLA/PCL/SiO_2_ blends.

PLA/PCL (wt%)	SiO_2_ (wt%)	*E* (MPa)	SD	*σ* (MPa)	SD	*ε_b_* (%)	SD	*W* (Nm)	SD
	0	1590.4	125.1	39.5	2.9	2.78	0.18	0.55	0.10
100/0	1	1619.2	61.7	50.5	4.6	4.50	0.34	1.31	0.15
	3	1741.6	59.7	50.5	11.6	3.74	1.32	1.02	0.52
	0	1513.2	89.3	45.2	2.5	5.85	3.48	1.13	0.30
90/10	1	1583.0	203.2	38.2	2.1	3.14	0.64	0.65	0.17
	3	1584.2	108.6	37.4	8.0	4.46	2.46	1.19	0.89
	0	1219.3	128.4	35.9	5.2	4.83	1.30	1.08	0.43
80/20	1	1379.5	84.3	31.0	4.6	2.59	0.17	0.40	0.17
	3	1335.1	80.7	32.4	1.2	7.37	0.67	1.28	0.23
	0	1019.9	172.5	31.2	3.0	5.37	1.77	0.91	0.37
70/30	1	1109.5	94.9	24.6	7.3	2.67	0.99	0.62	0.36
	3	1122.7	121.9	29.6	2.6	3.88	0.83	0.62	0.21
	0	972.2	82.8	27.9	3.6	6.30	2.53	1.02	0.42
60/40	1	1161.1	67.6	27.3	0.8	6.66	1.43	1.09	0.14
	3	1200.7	114.7	27.4	1.1	4.40	1.76	0.66	0.26
	0	932.4	28.5	23.6	1.7	4.68	0.80	0.63	0.18
50/50	1	929.1	68.1	26.7	0.7	8.70	1.75	1.36	0.18
	3	945.6	78.5	25.8	0.6	5.28	1.48	0.79	0.20

**Table 2:** Storage modulus (*E’*) in the glassy region at −80 °C and at room temperature 20 °C.

PLA/PCL (wt%)	SiO_2_ (wt%)	*E*’ at −80 °C (GPa)	*E*’ at 20 °C (GPa)
	0	5.374	2.541
100/0	1	4.976	2.558
	3	5.086	2.476
	0	6.920	2.561
90/10	1	4.672	1.788
	3	5.589	2.164
	0	6.736	2.589
80/20	1	5.490	2.007
	3	5.151	1.465
	0	4.905	1.312
70/30	1	3.869	0.8011
	3	5.251	1.119
	0	4.963	0.9030
60/40	1	4.642	1.155
	3	6.010	2.724
	0	5.016	1.009
50/50	1	5.698	1.250
	3	5.217	1.032

**Table 3:** DSC data for the second heating cycle of PCL/PLA/SiO_2_ blends.

PLA/PCL (wt%)	SiO_2_ (wt%)	PLA							PCL		
		*T_g_* (°C)	*T_cc1_* (°C)	Δ*H_cc1_* (J·g^−1^)	*T_cc2_* (°C)	Δ*H_cc2_* (J·g^−1^)	*T_m_* (°C)	Δ*H_m_* (J·g^−1^)	*T_g_* (°C)	*T_m_* (°C)	Δ*H_m_* (J·g^−1^)
100/0	0	60	99	32.1	154	2.4	169	−38.7	−	−	−
	1	59	97	27.9	154	3.8	169	−37.0	−	−	−
	3	59	100	28.2	156	2.9	170	−38.3	−	−	−
90/10	0	−	101	32.5	155	1.5	169	−34.0	−44	57	−2.7
	1	−	102	23.1	157	2.0	170	−29.0	−55	58	−2.2
	3	−	101	25.6	157	1.8	171	−31.4	−71	55	−1.2
80/20	0	−	100	22.4	159	1.1	168	−29.5	−	57	−7.0
	1	−	100	23.5	155	2.0	168	−27.7	−72	57	−5.3
	3	−	105	24.9	−	−	171	−29.4	−69	56	−3.2
70/30	0	−	100	19.0	155	1.2	168	−26.4	−72	57	−17.8
	1	−	101	17.9	156	0.9	170	−25.7	−68	57	−9.4
	3	−	107	22.2	−	−	171	−25.4	−64	56	−5.1
60/40	0	−	101	25.3	155	0.5	168	−23.9	−63	57	−16.6
	1	−	102	16.2	156	0.8	169	−20.1	−66	57	−15.3
	3	−	104	18.4	158	0.4	171	−21.1	−58	57	−14.1
50/50	0	−	102	14.3	156	0.5	169	−18.4	−62	57	−20.4
	1	−	102	12.8	156	1.8	173	−16.3	−67	57	−18.8
	3	−	102	14.6	158	1.3	172	−19.4	−56	64	−19.3

**Table 4:** DSC data for the cooling cycle of PCL/PLA/SiO_2_ blends.

PLA/PCL (wt%)	SiO_2_ (wt%)	PLA		PCL	
		*T_c_* (°C)	Δ*H_c_* (J·g^−1^)	*T_c_* (°C)	Δ*H_c_* (J·g^−1^)
100/0	0	89	26.4	−	−
	1	90	1.6	−	−
	3	92	2.3	−	−
90/10	0	87	28.2	27	3.5
	1	89	1.0	28	3.2
	3	90	0.4	23	3.1
80/20	0	83	9.4	26	7.9
	1	89	0.9	28	8.8
	3	93	0.2	26	6.5
70/30	0	/	/	28	15.4
	1	88	1.7	25	12.0
	3	93	0.4	24	11.8
60/40	0	90	1.0	26	17.9
	1	87	0.6	25	19.6
	3	90	0.6	25	16.3
50/50	0	116	0.8	26	25.1
	1	91	0.3	27	25.1
	3	90	1.1	25	22.3

**Table 5:** The degrees of crystallization of PLA (*X_c (PLA)_*) and PCL phase (*X_c (PCL)_*).

PLA/PCL (wt%)	SiO_2_ (wt%)	*X_c_* _(*PLA*)_ (%)	*X_c_* _(*PCL*)_ (%)
100/0	0	6.26	−
	1	8.65	−
	3	9.58	−
90/10	0	1.54	19.45
	1	6.11	7.97
	3	6.35	2.94
80/20	0	8.36	25.20
	1	4.94	18.99
	3	5.32	11.63
70/30	0	9.87	42.64
	1	10.65	22.57
	3	4.27	12.18
60/40	0	2.15	29.86
	1	6.18	27.37
	3	4.28	25.27
50/50	0	7.70	29.22
	1	6.47	26.92
	3	9.04	27.70

**Table 6:** Thermal degradation parameters of PLA/PCL blends.

PLA/PCL (wt%)	SiO_2_ (wt%)	*T_onset_* (°C)	*T_5%_* (°C)	*T_50%_* (°C)	*T_maxPLA_* (°C)	*T_maxPCL_* (°C)	*R_600 °C_* (%)
	0	312	316	345	349	−	1.3
100/0	1	299	298	341	349	−	1.0
	3	319	314	343	345	−	4.5
	0	291	281	341	341	372	0.7
70/30	1	323	321	357	354	395	0.9
	3	326	326	358	355	395	3.7
	0	303	306	352	335	380	0.5
50/50	1	327	325	370	351	400	1.5
	3	340	338	378	367	403	3.6

## Data Availability

Not applicable.
